# Intravenous levosimendan-norepinephrine combination during off-pump coronary artery bypass grafting in a hemodialysis patient with severe myocardial dysfunction

**DOI:** 10.1186/1749-8090-5-9

**Published:** 2010-03-02

**Authors:** Georgios Papadopoulos, Nikolaos G Baikoussis, Petros Tzimas, Stavros N Siminelakis, Menelaos Karanikolas

**Affiliations:** 1 Department of Clinical Anaesthesiology and Intensive Postoperative Care Unit, University of Ioannina School of Medicine, Ioannina, Greece; 2 Department of Cardiac Surgery, University of Ioannina School of Medicine, Ioannina, Greece; 3 Department of Anaesthesiology and Critical Care Medicine, University of Patras School of Medicine, Patras, Greece

## Abstract

This the case of a 63 year-old man with end-stage renal disease (on chronic hemodialysis), unstable angina and significantly impaired myocardial contractility with low left ventricular ejection fraction, who underwent off-pump one vessel coronary bypass surgery. Combined continuous levosimendan and norepinephrine infusion (at 0.07 μg/kg/min and 0.05 μg/kg/min respectively) started immediately after anesthesia induction and continued for 24 hours. The levosimendan/norepinephrine combination helped maintain an appropriate hemodynamic profile, thereby contributing to uneventful completion of surgery and postoperative hemodynamic stability. Although levosimendan is considered contraindicated in ESRD patients, this case report suggests that combined perioperative levosimendan/norepinephrine administration can be useful in carefully selected hemodialysis patients with impaired myocardial contractility and ongoing myocardial ischemia, who undergo off-pump myocardial revascularization surgery.

## Background

Levosimendan (OR 1259), the levo-isomer of racemic simendan [1] is a pharmacologic agent indicated for treatment of non-compensated heart failure. Levosimendan enhances myocardial contractility without increasing myocardial oxygen consumption [2,3] through two different mechanisms: (A) calcium-dependent binding to cardiac troponin C, thereby enhancing the calcium sensitivity of cardiac contractile proteins [3,4] and improving myocardial contractility, and (B) opening of ATP-dependent potassium channels in vascular smooth muscle, resulting in venous, arterial and coronary vasodilation [5-8], thereby reducing myocardial preload and afterload.

Levosimendan is 98% albumin-bound, its volume of distribution is 0.4 L/kg, plasma half life is 1 hour [9], and plasma clearance is 3 ml/kg/min [10,11]. Peak plasma concentrations occur 12 minutes after a bolus dose or 4 hours after starting a continuous infusion without a bolus. Levosimendan is extensively metabolized in the liver, is eliminated mainly by conjugation and excretion in urine and feces, and its elimination half-life is 1 hour [12]. After IV levosimendan administration, 5% of the drug is reduced in the small bowel to OR-1855, which is reabsorbed to the systemic circulation, and is then metabolized to OR-1896, which is pharmacologically active and produces a hemodynamic profile comparable to the parent-drug. OR-1896 is only 40% protein-bound, its peak plasma concentration is observed 1-4 days after levosimendan infusion ends [10], its half life is 80 hours, and it is responsible for the extended (7-9 days) duration of levosimendan clinical action [5,12-14].

Levosimendan is not dialyzable. In contrast, OR-1855 and OR-1896 are dialyzable, but their dialysis clearance is very slow (8-23 ml/minute). Consequently, the net effect of a 4-hour hemodialysis session on exposure to active metabolites is limited [3], and the AUCs for OR-1855 and OR-1896 are increased by 170% in hemodialysis patients. Although the Levosimendan package insert [3] states that levosimendan should not be used in ESRD patients, there is one case report of postoperative use [13], but no reports of intraoperative levosimendan use in hemodialysis patients. In this case report we describe a hemodialysis patient with severe CAD, ongoing myocardial ischemia despite maximal medical therapy, low LVEF and severe bilateral ICA stenosis. The patient received a 24-hour continuous IV levosimendan/norepinephrine infusion during and after OPCAB surgery, with very satisfactory results: myocardial contractility, CO, CI, SvO_2 _and INVOS markedly improved, and there was no hypotension or exacerbation of myocardial ischemia.

## Case presentation

A 63 year-old Caucasian man with unstable angina and chronic renal failure underwent one vessel OPCAB. Past medical history included smoking 2 packs per day for 40 years, hypertension, IDDM treated with insulin for 15 years, PVD with claudication and bilateral ICA stenosis, ESRD (Cr: 8.4 mg/dL, BUN: 199 mg/dL) which had been attributed to long-standing poorly controlled hypertension, and was treated with periodic (every other day) hemodialysis for 6 years, and sick sinus syndrome. He had a pacemaker (programmed in DDD mode with baseline HR set at 60/minute) inserted three years before this myocardial revascularization procedure, but the pacemaker was turned off immediately after anesthesia induction. He also had intermittent claudication, with preoperative angiography revealing significant right iliac and left femoral artery stenosis, extensive abdominal aorta calcification and 80% bilateral ICA stenosis. Coronary angiography revealed 3-vessel disease, with 80% mid-LAD stenosis, complete proximal and distal LCX occlusion with retrograde filling from the LAD, and complete ostial RCA occlusion. Transthoracic echocardiography revealed LV dilatation with akinetic basal inferior and basal posterior LV wall, hypokinetic medial posterior LV wall, LVEF estimated at 25-30%, moderate mitral regurgitation and pulmonary hypertension (estimated peak PA pressure 58 mmHg). In the last week before surgery, the patient had difficulty completing hemodialysis sessions due to serious hypotension, and experienced unstable angina, while under maximal medical therapy with nitrates (transdermal glyceryl trinitrate 10 mg per 24 hours), ACE inhibitors (p.o. enalapril 10 mg per day) and aspirin (p.o. 325 mg per day). Because of his unstable condition, we decided to proceed with myocardial revascularization only, and consider surgical treatment of bilateral ICA stenosis later. Furthermore, we chose the OPCAB technique, in order to lower the risk of adverse cerebral events and avoid the undesirable consequences of cardiopulmonary bypass. Monitoring included, in addition to the standard monitors mandated by the American Society of Anesthesiologists, invasive blood pressure through a right radial arterial line, CVP, PA and PAOP pressures through a PA catheter, which was inserted immediately after induction of anesthesia and was removed on the 2^nd ^postoperative day. We also used INVOS (Cerebral Oximeter System, Somanetics), with sensors attached to the patient's forehead, to monitor adequacy of cerebral perfusion, and TEE to monitor myocardial contractility. Anesthesia induction was uneventful, without any hemodynamic derangement. Mean arterial pressure was maintained at 60 mmHg or higher, while the PA catheter revealed pulmonary hypertension (SPAP: 56 mmHg, PAOP: 18 mmHg, CVP: 18 mmHg, CO: 2.8 L/min, CI: 1.6 L/m^2^/min SvO_2 _49%, SVR 1114). As direct visualization of the heart confirmed severely impaired myocardial contractility with abnormal distension of both ventricles, we decided to start inotropic support using a combined levosimendan/norepinephrine infusion in an attempt to improve myocardial function and increase cardiac output, while avoiding myocardial ischemia and hypotension. The decision to use a levosimendan/norepinephrine combination was based on the need to (A) improve myocardial contractility and cardiac output without increasing myocardial oxygen consumption, and (B) avoid hypotension, which could aggravate myocardial and brain ischemia. Because of hypoalbuminemia, we started IV levosimendan infusion at only 0.07 μg/kg/min, while IV norepinephrine infusion started at 0.05 μg/kg/min and was titrated to effect. Baseline INVOS values were very low before anesthesia induction (42 on the left, 37 on the right side) and increased only slightly after anesthesia induction (45 on the left, 43 on the right side). However, thirty minutes after levosimendan infusion started, CO, CI and SvO_2 _improved significantly (to 3.6 L/min, 2.1 L/m^2^/min and 70% respectively), LVEF increased to 50% and INVOS also increased significantly (to 59 on the left, 53 on the right). Despite the need to gradually increase norepinephrine dose to 0.15 μg/kg/min, in order to maintain MAP >60 mmHg, CI and SvO2 continued to rise, while CVP, PA and PAOP declined slightly over the ensuing 3 hours (while surgery was still underway), and this improvement persisted during the entire postoperative period (table 1). Accidental intraoperative rupture of the very thin anterior RV wall resulted in hemodynamic collapse, requiring prompt, rapid administration of 5 units of red blood cells, 2 units of FFP and addition of epinephrine infusion at 0.07 μg/kg/min. Hypotension lasted approximately 30 minutes, until the RV wall rupture was securely corrected (without requiring extracorporeal circulation). Postoperatively, the patient was transferred to the ICU, where levosimendan infusion continued for 24 hours and norepinephrine continued for 40 hours. The patient was extubated on POD 1, and had uneventful hemodialysis a few hours after extubation. Detailed postoperative neurologic examination did not reveal any neurologic deficits. Vital signs remained stable postoperatively, except for an episode of hypotension shortly after hemodialysis on POD 5. This hypotensive event resolved with volume loading, and was attributed to pericardial effusion, which delayed discharge from the hospital until POD 12. Three months later, the patient was in good condition, had a normal life and continued hemodialysis three times/week without any problems. Follow-up echocardiography 4 months after the operation showed somewhat improved myocardial contractility, with LVEF estimated at 40%, mild mitral regurgitation and estimated peak pulmonary artery pressure at 35 mmHg. Now, three years later, he is still alive and doing remarkably well.

**Table 1:** Hemodynamic and INVOS data

	T_1_	T_2_	T_3_	T_4_	T_5_	T_6_	T_7_	T_8_	T_9_	T_10_
HR	60	62	64	64	61	64	65	66	63	68
P_syst_	100	125	104	113	108	97	101	110	91	106
P_diast_	45	71	51	61	58	52	51	58	48	55
MAP	57	91	61	80	76	69	70	83	67	70
CVP	18	18	17	16	16	14	15	12	10	10
PAP_syst_	56	48	46	46	44	44	40	39	31	35
PAP_diast_	25	18	17	17	17	19	20	17	19	17
PAP_mean_	37	30	30	30	25	25	26	23	24	24
PAOP		18	17	16	16	16	16	16	16	15
CO	2.8	3.6	4.1	4.2	4.0	4.6	4.3	4.6	4.6	4.6
CI	1.6	2.1	2.4	2.4	2.0	2.7	2.5	2.7	2.7	2.7
SVR	1114	1622	858	1219	1200	956	1023	1234	991	1043
PVR		266	253	266	180	156	186	121	139	156
LVEF	25			40	40					
SvO_2_	49	70	80	80	82	82	80	60	65	58
INVOS_Left_	45	59	59	55	60	60				
FiO_2_	0.5	0.5	0.5	0.5	0.5	0.6	0.6	0.4	0.4	0.21
Norepinephrine*		0.05	0.08	0.12	0.15	0.12	0.15	0.2	0.08	0.02

* Dose in μg/kg/min

HR in beats/minute

P_syst, _P_diast, _P _mean, _CVP, PAP_syst, _PAP_diast, _PAP_mean, _PCWP, all measured in mmHg

CO = L/min, CI = L/m^2^/min S_V_O_2 _= %, INVOS = %

T_1 _before levosimendan/norepinephrine infusion started

T_2 _1 h after levosimendan infusion started

T_3_, T_4 _2 and 3 hours after levosimendan infusion started

T_5 _at end of surgery

T_6 _T_7 _6 and 12 hours after surgery

T_8, _T_9, _T_10 _18, 24, 36 hours after surgery

## Conclusions

Levosimendan is a newer therapeutic agent for treatment of cardiac failure [13], is generally well tolerated, and its main side effects are usually due to vasodilation. Although levosimendan has been administered to patients with mild to moderate renal disease without serious adverse consequences [14], we could find only one published case of postoperative (but not intraoperative) levosimendan administration in a hemodialysis patient [13]. Despite the absence of published data, we decided to use levosimendan in our patient, because he had significantly impaired LV function, low CO, CI and SvO_2_, and evidence of impaired cerebral oxygenation, as measured by INVOS. We therefore needed to improve myocardial contractility, CO, CI and SvO_2 _without increasing myocardial oxygen consumption and without hypotension, which could be detrimental, due to severe bilateral ICA stenosis. Under the circumstances, combined levosimendan/norepinephrine use was a reasonable choice: levosimendan improves myocardial contractility without increasing myocardial oxygen consumption [15-17], while norepinephrine has desirable inotropic and vasopressor properties. Use of IABP could also be a reasonable option, but insertion of IABP in this particular patient would be problematic due to extensive peripheral arterial (aortic, iliac and femoral) calcification and stenosis, and could further compromise lower extremity circulation. Dobutamine, milrinone and/or epinephrine could also improve myocardial contractility, but would likely increase myocardial oxygen consumption, and thereby exacerbate myocardial ischemia. In our case, the levosimendan/norepineprhine combination worked as predicted, conferred significant hemodynamic improvement (despite unexpected surgical complications necessitating rapid intraoperative RBC and FFP transfusion) and facilitated completion of the OPCAB procedure without need for extracorporeal circulation. Thus the patient benefitted from improved myocardial contractility, increased CO, CI and SvO_2 _and reduced CVP and PAOP, and these beneficial changes lasted for several days after levosimendan infusion stopped. We believe that the effectiveness of levosimendan at this low dose (0.07 μg/kg/min) was due to reduced protein binding because of hypoalbuminemia (albumin plasma level was 3.1 mg/dL in this case). In addition, the use of a low levosimendan dose, the combination with norepinephrine and close monitoring, in an attempt to avoid or promptly treat hypotension, all likely contributed to hemodynamic stability in this case. In conclusion, this case report suggests that combined levosimedan/norepinephrine IV infusion is a reasonable inotropic support option in patients with heart failure and ongoing myocardial ischemia, even in the presence of end-stage renal disease and severe bilateral internal carotid artery stenosis.

## Consent

Written informed consent was obtained from the patient for publication of this report. A copy of the written consent is available for review by the Editor-in-Chief of this journal.

## Abbreviations

ACE inhibitors: Angiotensin-Converting Enzyme Inhibitors; ATP: Adenosine Tri-Phospate; CABG: Coronary Artery Bypass Grafting; CAD: Coronary Artery Disease; CI: Cardiac Index; CO: Cardiac Output; CRF: Chronic Renal Failure; CVP: Central Venous Pressure; ESRD: End-Stage Renal Disease; FFP: Fresh Frozen Plasma; HR: Heart Rate; IABP: Intra-Aortic Balloon Pump; ICA: Internal Carotid Artery; IDDM: Insulin-Dependent Diabetes Mellitus; INVOS: IN Vivo Optical Spectroscopy; IV: Intravenous; LAD: Left Anterior Descending; LCX: Left Circumflex Coronary Artery; LV: Left Ventricle; LVEF: Left-ventricular ejection fraction; MAP: Mean Arterial Pressure; OPCAB: Off pump coronary artery by-pass; PA: Pulmonary Artery; PAOP: Pulmonary Artery Occlusion Pressure; POD: Postoperative Day; PVD: Peripheral Vascular Disease; RBC: Red Blood Cells; RCA: Right Coronary Artery; RV: Right Ventricle; SvO_2_: Mixed Venous Oxygen Saturation; TEE: Trans-Esophageal Echocardiography.

## Competing interests

This work was supported solely by department funds. All authors declare that they have no competing interests.

## Authors' contributions

GP supervised intraoperative and postoperative anesthesia care, conceived the study and revised manuscript, NB assisted with the operation, participated in postoperative patient care and collected data, PT provided intraoperative and postoperative anesthesia care and collected data, SN performed the operation, directed postoperative care and revised manuscript, MK did data interpretation, wrote and revised manuscript. All authors have read and approved the final manuscript.
